# Living infodemics across borders: experiences during the COVID-19 pandemic among migrants from India living in Norway

**DOI:** 10.3389/fpubh.2025.1488080

**Published:** 2025-02-28

**Authors:** Prabhjot Kour, Sai Harish Adari, Bernadette Kumar, Esperanza Diaz

**Affiliations:** ^1^Pandemic Centre, Department of Global Public Health and Primary Care, University of Bergen, Bergen, Norway; ^2^Division for Health Services - Management and Staff, Norwegian Institute of Public Health, Oslo, Norway; ^3^Department for Health and Function, Western Norway University of Applied Sciences, Bergen, Norway

**Keywords:** infodemic, Indian migrants, COVID-19, transnationalism, Norway

## Abstract

During the COVID-19 pandemic, migrants living in Norway navigated a complex “infodemic”, which encompassed diverse health information sources from health authorities and media from both local and their home country. This study aimed to explore the experiences of Indian migrants in Norway related to their encounters with varied, and sometimes inaccurate and conflicting information between India and Norway amidst COVID-19 pandemic. Qualitative interviews were conducted with 12 Indian migrants and data was analyzed using thematic analysis. The analysis yielded five main themes: (1) Transnational sources of information, (2) Drivers for trusting information, (3) Transnational healthcare-seeking behavior, (4) Influences underlying decision-making, and (5) Emotional impact of conflicting information. Our findings highlight the complexities of information consumption and its effects on the cognitive-emotional processes of migrants, discussing the role of factors such as familiarity, emotional ties, and trust. Further, the study identified the need for culturally sensitive health communication interventions, the establishment of trust with migrant communities through accurate information dissemination, and the requirement of strategies to support the emotional well-being of migrants in situations when health information varies quickly and differ geographically. The implications of this research extend to the development of effective public health strategies for migrant communities during global health crises.

## 1 Introduction

The Coronavirus disease (COVID-19) was the first pandemic in history where technology and social media were extensively used to keep people safe, informed, and connected (1). However, the same technology that maintained our connection and flow of information also fueled and amplified an “infodemic” which undermined the global response and jeopardized efforts to control the virus. According to the World Health Organization (WHO), the COVID-19 pandemic and its response were accompanied by *a massive infodemic: an overabundance of information—some accurate and some not—that makes it hard for people to find trustworthy sources and reliable guidance when they need it* (1). Further, infodemic included deliberate attempts to spread the wrong information in the form of mis- and dis-information or conspiracy theories, amplified through social networks, both online and offline. This sort of information has harmful impact on people's physical and mental health, promote hate speech and increases stigma contributing to exclusion of vulnerable populations including migrants (2). In turn, this affects individual's decision-making processes, reduces trust in health institutions and results in inadequate adherence to public health measures, undermining countries' efforts to curb the pandemic (1, 3).

The negative impacts of the infodemic might be more severe in low and middle-income countries with inadequate healthcare infrastructure, limited resources, and low health literacy rate (2), and it also affects migrants in high-income countries (4–7). Islam et al., identified reports on COVID-19 related rumors, stigma and conspiracy theories and impact on health in 25 languages from 87 countries. They reported that 82% of the claims with text ratings were false, with topics ranging from illness, transmission and control measures to causes, treatment and cure (8). Wrong information regarding similar topics related to COVID-19 infodemic has also been reported among migrants living in high-income countries such as Norway (4), Portugal (5), the United Kingdom (6), and the United States (7).

Navigating health information effectively in times of health crises is a complex endeavor. Individuals, including migrants, need health literacy to access, understand, integrate and use health information, as well as to communicate clearly with their healthcare providers to be able to take well informed health decisions. The term health literacy includes both personal and organizational health literacy. Personal health literacy refers to how individuals comprehend and act on the health information for themselves and others, whereas organizational health literacy focuses on how well institutions provide accessible and understandable health information and services. Additionally, digital health literacy (DHL), became a critical skill during COVID-19 pandemic for all the groups of society, including migrants. It is defined as the ability of people to obtain, understand, and assess health information from digital technologies, including the skills required to navigate online platforms, evaluate the reliability of health information, and apply the acquired knowledge to make health-related decisions (9). For clarity and simplicity in this paper, we will use the term “health literacy” to collectively refer to personal, organizational and digital health literacy.

Health literacy is crucial in an era marked by an infodemic, where distinguishing between true and false information is pivotal for making health related decisions. Language barriers, lack of attention to cultural factors and the challenges of adapting to new healthcare systems profoundly impair migrants' ability to understand and utilize health information effectively (10, 11). A study conducted in Norway during COVID-19 pandemic, on five migrant groups reported lower health literacy as compared to the general population, and it varied with factors such as age, level of education attainment, economic status, and long-term illness. Further, the study reported migrant's willingness to use digital health services was linked to these factors (12). A scoping review on health literacy among migrants during the first 2 years of the COVID-19 pandemic found similar results. It further highlighted that media, family, peers, and community networks play crucial roles in shaping health literacy of migrants, especially when they distrusted governmental information and relied more on their personal networks (13).

Personal networks across borders are particularly important for migrants, making transnationalism emerge as a critical factor shaping their health literacy. I this setting, transnationalism, characterized by ongoing social, economic, and cultural ties maintained by migrants with their countries of origin and other diasporic communities, plays a pivotal role in influencing health literacy (14). During pandemic, the term “pandemic transnationalism” was coined referring to “circulation of ideas and practices in times of pandemic which encompasses an exchange of informal practices that affects not only the lives of migrants and their families in their home country, but their close circle of friends and neighbors as well” (15). Studies have reported that migrants engaged in new conversations through transnational social networks, where they encountered diverse and sometimes conflicting information, including varying opinions on issues like hygiene, healthcare and contagiousness of disease as well as acceptable social behavior (15, 16). These interactions exposed migrants to navigate through varied cultural perspectives and caregiving practices, reflecting differences in countries' pandemic strategies and contributing to challenges and tensions (14).

Migrants from India living in Norway exemplified these challenges and tensions as they navigated between the pandemic transnationalism because India and Norway faced very distinct situations and information challenges during the COVID-19 pandemic (17, 18). Norway required extensive translation and interpretation services to address communicative barriers among various migrant groups (17), while India grappled with a high volume of misinformation with the surge of digital solutions, leading to the unregulated use of self-medication and particularly increased the vulnerability among those with underlying chronic conditions, such as diabetes (18).

Of the migrant population, around 25,000 are from India (19). The Indian migration to Norway began in early 1970s, driven by the search for better economic opportunities and educational prospects. Today, the Indian community in Norway is diverse, encompassing professionals in IT, medicine and engineering, as well as business owners and students. They are recognized for their socio-economic and cultural integration, maintaining strong ties with India while contributing to both countries' economic and cultural exchange (20). However, they face health challenges, with a higher prevalence of chronic diseases such as diabetes compared to the general Norwegian population, reflecting trends observed in Indian communities globally (21, 22). During the COVID-19 pandemic, Indian migrants in Norway faced distinct health challenges, with lower COVID-19 vaccination rates (88%) compared to the overall population (93%) (23), highlighting disparities in healthcare access and management.

While much research has explored the challenges faced by migrants in Norway during the COVID-19 pandemic, there is a significant gap in understanding specifically how the differences in health-related information from home and host countries, such as India and Norway, influence migrants' decision-making. Limited studies focus on how migrants navigate and assess conflicting information and determine which sources to trust. Given the differences in COVID-19 prevalence and health practices between India and Norway, targeted research on Indian migrants in Norway presents an outstanding opportunity to shed light on how specific migrant groups manage health crises and infodemics during pandemics. This study aimed to explore the experiences of Indian migrants living in Norway, of how being exposed to infodemic, especially with transnational information sources, affected their risk perceptions and decision-making regarding protective behaviors about COVID-19 pandemic.

## 2 Methods

### 2.1 Study design

The study employed an explorative and descriptive qualitative design to explore how Indian migrants living in Norway perceived and managed exposure to information sources regarding protective behaviors and risk perceptions during the COVID-19 pandemic. Utilizing semi-structured interviews, the study aimed to explore participants' views, particularly regarding their experiences with information from both India and Norway.

### 2.2 Recruitment

A criterion-based, purposive sampling method followed by snowballing was employed to recruit the participants. The inclusion criteria were persons with migrant background who were born in India to Indian parents, currently residing in Norway and were in Norway during the pandemic, and above the age of 18. Migrants in our study are understood as persons born abroad of two foreign-born parents and four foreign-born grandparents and they contribute to 16.8% of Norwegian population (19).

The recruitment process for Indian migrants in Norway was meticulously designed to ensure the inclusion of a diverse group of participants in terms of age, gender, educational backgrounds, and length of stay in Norway. Various strategies were adopted to identify individuals who could contribute valuable insights to this study. One of the primary methods employed for participant identification involved community engagement. Prospective participants were approached by second author, during community events and gatherings attended by the Indian migrant community in Bergen. These occasions afforded an opportunity to interact with individuals actively engaged in community activities. Moreover, informal exchanges during weekend recreational activities facilitated the identification of individuals who might not have participated in formal community gatherings. Recognizing the importance of including participants with extensive experience living in Norway, additional steps were taken to identify some individuals who had resided in the country for longer duration. Established business owners and retired individuals were identified and engaged in this study and their long-standing presence in Norway provided valuable insights into the evolution of their COVID-19 experiences and information-seeking behavior. This approach aimed to capture a wide range of perspectives and experiences within the Indian migrant population in Norway.

A total of 12 participants were recruited and interviewed for this study.

### 2.3 Participants

Twelve participants (Appendix 1) accepted to be interviewed for the study. At the time of the interview, the age range was between 20 and 79 years; all were married and had some level of formal education. There were 4 females and 8 males. The length of residence in Norway among participants varied between 3 and 46 years.

### 2.4 Data collection

Data was collected through eight individual (24) and two dyadic (conducted with two participants) (25) interviews between August 2023 to October 2023. The interviews were conducted by the second author in a quiet setting, chosen based on each participant's preference to facilitate open and honest communication. Each interview lasted between 30 and 40 min. An interview guide (Appendix 2) was created and agreed upon by second, third and fourth author, consisted of open-ended questions to explore the explore their experiences with various transnational information sources and how these shaped their protective behaviors and risk perceptions amid the COVID-19 pandemic. Questions ranged from the types of information sources consulted to their strategies for assessing credibility and the influence of their transnational background on these processes. All the interviews were conducted in English, audio recorded and transcribed verbatim.

Audio recordings of the interviews were deleted after the transcription. The interview transcripts were securely stored on a protected server, SAFE, without the direct personal identifying information of the participants. All the participants were assigned numbers, which are used in the presentation of the results below.

### 2.5 Data analysis

The interview transcripts were read and analyzed using thematic analysis (26). The explicit aim of the analysis was to develop new descriptions by directly presenting the experiences of the participants as expressed by them rather than exploring the possible underlying meanings behind their statements, that is, on a semantic level. The following steps of thematic analysis were adopted: firstly, the transcripts were read several times for familiarization with the data; secondly, the data was coded in a systematic fashion across the entire dataset, generating initial codes and collating data relevant to each code. After coding and collating the data, the codes were sorted into potential themes and were reviewed. Following this process, the entire data was read again, and themes were adjusted based on the aim of the study. Lastly, a thorough review was conducted for each theme, by systematically arranging the data extracts with accompanying quotes, constituting the results section of this paper. The initial analysis was performed by the first author and agreed upon by all authors in the subsequent stages.

### 2.6 Ethical considerations

Following an ethical assessment, the study was registered in the Norwegian Agency for Shared Services in Education and Research (SIKT) with reference number 217472. The research procedure adhered to the principles outlined in the Declaration of Helsinki. Participation in the study was voluntary, and measures were taken to safeguard the confidentiality and anonymity of the participants. Informed consent was taken, and it was made sure that all the participants were completely aware of the purpose of the study and could withdraw at any point in time.

## 3 Results

The analysis yielded five themes in which participants described their experiences of how the transnational information from India was perceived and used to respond to COVID-19 related risks in Norway. These themes were: (1) Transnational sources of information, (2) Drivers for trusting information, (3) Transnational healthcare-seeking behavior, (4) Influences underlying decision-making, and (5) Emotional impact of conflicting information.

### 3.1 Transnational sources of information*: “where I seek out information”*

Participants highlighted the different sources that they used to seek the information both in India and Norway, which included international agencies such as WHO, official national governmental websites and apps, newspapers, national news channels, social media and messaging apps like WhatsApp. The consumption of the information was in multiple languages, such as English, Norwegian and various Indian languages. Most of the participants sought information from digital platforms, however traditional sources such as newspapers were also discussed as the source of information.

*Yes, I primarily relied on news channels for updates. I also followed newspapers*. P4

*I just Googled it. I'll follow that because I don't know what is the most trustable one*. P1

Further, younger participants stated their preference of using social media and messaging apps for their simplistic usage, quick updates and sense of connection they provided with their families and friends in India. This preference of information source was linked to sense of belonging during the uncertainties of pandemic, serving both as a coping mechanism and a possible cause of stress due to spread of misinformation.

*Initially I didn't rely on Facebook, social media or friends for information. However, after some negative experiences within my family (citing COVID-19 related illness and deaths) I started paying more attention to the news and various sources including YouTube, Facebook and WhatsApp*. P6

*I think in terms of getting information regarding COVID I was of course reading newspapers and then also relying on social media channels… I was concerned about India like how the situation is there*. P5

### 3.2 Drivers for trusting information: “*finding trust in familiar voices”*

Participants described the various drivers based on which they chose to trust the information, integrating both local and international cues. Most participants showed a clear preference for verified and official sources from both India and Norway to gather information during the COVID-19 pandemic. Their behaviors and decisions indicated a deliberate and careful method of consuming information. They consistently mentioned their trust in authoritative sources like the WHO, suggesting a cautious approach amidst the abundance of unreliable information during the global health crisis. This reliance on credible sources seems to reflect their need for accurate information in a time marked by uncertainty and widespread misinformation.

*I think I would go for WHO because that might give the best information since it is the World Health Organization and so I trusted that most*. P1

*I go with the government sources (referring to Norway), published by the corporations, the municipality. Because it was based on trust, or they must have done due diligence to verify that the information is correct, and they then released it to the public. I think whenever I get information and if I didn't feel like it's trustable, I just went online and looked for genuine sources like the BBC or you know the other trusted media and then read upon it*. P3

It was also stated by the participants that they consider the personal experiences shared by close friends, family and neighbors (both in India and Norway), through direct conversations, as a trustful source of information. This in turn helped them to tackle with the rumors and misinformation that were circulated enormously through social media or messaging apps, as narrated by one participant:

*I learned about people's experiences with COVID-19 through direct conversations and not social media or WhatsApp. I think I trust the experiences shared by individuals rather than relying on rumors. I believe they provide more credible information, and I find them trustworthy*. P1

*Absolutely, family recommendations from India played a significant role for us*. P2

Further, the older participants trusted traditional media due to its established credibility and journalistic integrity. This choice was shaped by prior experiences in India, where traditional media has been a longstanding pillar of information. During the COVID-19 pandemic, this older group considered local Norwegian news outlets to be the most reliable source of information owing to rigorous fact-checking of the abundant information they came across.

*I was of course reading newspapers and then also relying on social media channels. I trusted like the (Norwegian) national newspapers or the news channels, which were there also on social media. That was something that I had been mostly seeking information*. P5

*Helse Norge (official website) provided accurate and reliable information on a day-to-day basis. I found the information from Helse Norge and Bergen municipality very helpful and easy to understand*. P2

### 3.3 Transnational healthcare-seeking behavior: “*I am still consulting my Indian doctor despite meeting my GP here*”

Some of the participants highlighted the role of healthcare professionals in India for seeking information related to COVID-19 symptoms or vaccination. Thereby stating the active engagement with transnational healthcare network, which influenced their healthcare seeking and decision making in Norway. This healthcare-seeking behavior includes seeking for information and was grounded on the existing relationships and ongoing treatments for medical conditions unrelated to COVID-19. Further, participants mentioned comparing the medical information between India and Norway, in order to make sense of the COVID-19 situation through their understanding.

*I felt mentally and physically drained, and I needed someone I could rely on. That's why I turned to my Indian doctor because I kept explaining to the doctors here that all these issues started after I had COVID and received the vaccination. I trust my Indian doctor more. They are more familiar with my medical history and conditions compared to my GP here (Norway)*. P6

*I am a bit shy to talk about very personal things with my local GP in Norway. But I am not shy to speak with my Indian doctor because he knows me better*. P2

Further, comparing between healthcare systems of Norway and their home country shaped the participants' perceptions of the efficacy and responsiveness of Norwegian health authorities. As a result, many expressed increased satisfaction and adherence to the guidelines provided by these authorities. However, the narrative wasn't uniformly positive. One participant highlighted specific challenges faced as a migrant, particularly in terms of accessing healthcare services, communicating effectively, and seeking guidance with local providers in Norway. These challenges weren't directly attributed to the COVID-19 pandemic, but the participants felt that such issues are a common aspect in their experience as migrant, indicating barriers in healthcare accessibility and communication.

*Language is a significant barrier. I find it easier to explain my health issues in my native language. Moreover, there is a sense of comfort and familiarity with my Indian doctor who understands my cultural background and my medical history. This familiarity makes it easier to discuss personal health matters. I am a bit shy to talk with the local GP about very personal things. But I am not shy to speak with my Indian doctor because he knows me better*. P2

*I used to call my family doctor in India. We generally don't get direct contact with the doctor in Norway through phone. If there is some emergency, then we need to make an appointment to go there. So, I used to call my family doctor back in India*. P7

### 3.4 Influences underlying decision-making: “*the need for travel, we had no other option*”

Experiences related to vaccination were used as an example of decision making in the interviews. Participants expressed varying attitudes toward COVID-19 vaccination. Some participants were driven to get vaccinated more by regulatory requirements than by a personal belief in the efficacy of vaccines. The strict implementations in place by local authorities, workplaces, or travel requirements were influential factors in their decision-making process. Concerns were raised about conflicting information and uncertainties, particularly regarding the perceptions of vaccination efficacy and potential side effects as social media has contrasting information.

*We were unsure about the reliability of the vaccines, which made us hesitant. We took our time before deciding to get vaccinated. There were various rumors on social media about the vaccines causing blood clots and other side effects*. P10

*My decision to get vaccinated was influenced by travel requirements, as it was mandatory to have vaccination reports for travel…I didn't have an option. So, it was not because I trusted that vaccine would work. It was just purely based on convenience*. P3

Transnational information sources also played a role, especially for those who consulted with family or medical professionals in India before deciding on vaccination. One participant had also discussed about his conversations within his professional networks in Norway to mitigate fears and skepticism around vaccine uptake, while another participant mentioned the need of social interactions after being isolated during the pandemic as an important factor for taking vaccination.

*We received constant reminders about our vaccination appointments, and since we plan to stay here for several more years, it made sense to get vaccinated*. P6

*If none of the restrictions were in place, I will not take any vaccine. Because I know my DNA, and my immunity is good enough*. P2

The decision-making process regarding vaccination among participants was multifaceted, influenced by the interplay of various factors such as regulatory pressure (mandatory for travel across nations) personal health belief, transnational consultations and discussions at workplace.

### 3.5 Emotional impact of conflicting information*: “It felt like never-ending”*

All the participants mentioned the emotional impact of COVID-19, often amplified by conflicting transnational information, and described considerable mental health challenges. Uncertainties around vaccination along with ongoing pandemic concerns, anxiety about long term effects of COVID-19 and aftereffects of COVID-19 vaccine were addressed by the participants. The emotional impact was also evident in worries about family members in their home country especially when considering the well-being of older adults family members back in India.

*After vaccination my health hasn't been stable. I developed thyroid issues which I didn't have before. My hormone levels became imbalanced, and my health has been a concern*. P4

*Many got infected in India and my parents were among them, and I was very much concerned about that*. P2

Participants also expressed confusion and anxiety arising from approaches to medication following a COVID-19 diagnosis. One participant, with an overview insight from both India and Norway, discussed contrast in medical practices. In India, a defined list of medications to be used for COVID-19 disease made him feel that it provided a structured approach which is also endorsed by healthcare professionals. However, in Norway, a notable absence of prescribed medicines left him with uncertainty. These contrasting approaches created a challenging emotional state.

*Another big source of confusion was the medicines that one should take once you get COVID. In India and Norway, there were completely different approaches to this question. In India, there was a list of medicines that was being used by the people, even prescribed by the medical practitioners. However, in Norway, no medicines were prescribed. As someone who had information from both the locations, it was not clear what to trust*. P8

Further, participants expressed anxiety about the seemingly endless nature of the pandemic and its potential long-term presence in society. Transnational networks were not limiting as coping mechanism but also a source of emotional stress due to conflicting information, which was described by participants as a never-ending situation.

*I think I was more concerned about what they said during the first wave and probably during second wave but at some point, after second wave or during I stopped following news on this because it was not helping me with anything. It was just creating more chaos. It felt probably COVID is never going to get over*. P5

*I used to ignore Facebook and social media because sometimes I feel that those are not relevant information as they might exaggerate the number and can cause confusion and stress*. P7

## 4 Discussion

Among Indian migrants living in Norway, this study explored the experiences of being exposed to transnational sources of information and how this shaped their risk perceptions and decision making regarding protective behaviors during the COVID-19 infodemic. Participants described various transnational sources they used to inform themselves, what made them trust the information and influenced their decision -making. Further, participants described their healthcare-seeking behavior both in Norway and India, and the emotional implications related to the COVID-19 pandemic.

In our study, participants described using a vast array of information sources during the COVID-19 pandemic consistent with previous studies conducted in Finland (27) and Norway (28), where migrants stated using a blend of messaging apps, social media, news outlet and interpersonal connections transnationally to stay informed.

The perceived reliability and trustworthiness of information sources are key to help individuals to navigate and make informed health-related decisions (29), especially in terms of infodemic. In our study, most participants demonstrated a clear belief that the official information sources were trustworthy and accurate, and accordingly deliberately chose these trusted sources to get authentic information. However, older participants preferred using Indian traditional media due to its established trustworthiness through earlier experiences in India, where traditional media has been a longstanding pillar of information. This may also be influenced by lower digital health literacy in this group, and hence limited use of information seeking channels. Similar findings have also been reported in a European study, where older migrants trusted traditional media more than social media, indicating the influence of old age and its intersection with cultural background on the trustworthiness of the source (30).

Health literacy has a crucial role in evaluating the trustworthiness of the source of information and is influenced by cultural background (10, 11). In our study, participants' trust in the information was dependent on the cross-verification using several transnational official sources, reflecting high health literacy skills in interpreting and evaluating the sources. This may further be influenced by previous history of information seeking behavior and trustworthiness, both in the country of origin and in the host country. Within this regard for the host country, a Norwegian study revealed that migrants' trust in authorities during COVID-19 pandemic was significantly shaped by local connections (with individuals, groups and entities) formed within Norway, expanding the understanding that trust is not solely influenced by cultural factors but importantly by interpersonal live experiences in the new country (31), similar to what our participants described. In parallel, trust in Indian official authorities during COVID-19, was bolstered by the proactive communication strategies employed by the Indian Council of Medical Research, which included disseminating detailed information and countering rumors as they appeared in India (32). This aligns with our study's finding that trust in authorities is enhanced by their targeting of specific and relevant questions and underscores the importance of collaboration of official sources with immigrant communities in host countries.

Participants in our study demonstrated trust in the Norwegian authorities by relying on their recommendations and efficacy in line with previous Norwegian study (31), while maintaining their trust in Indian doctors based on their previously established personal relations, especially when contact with Norwegian health professionals was not perceived as adequate. Nevertheless, most participants stated that they sought healthcare guidance from both, Norwegian GP and Indian family doctors, demonstrating the ability and skills to understand and navigate through two different healthcare systems. This can be further understood through the lens of transnationalism, where migrants sustain their connections with their home countries while living in abroad (33). These transnational linkages have significant impact on different aspects of migrants' behavior (including, information and healthcare-seeking) and decision-making, suggesting the endurance to their roots even during a global health crisis (33).

The COVID-19 infodemic across Norway and India was particularly associated with COVID-19 vaccination uptake. Despite, all our study participants were vaccinated, they still expressed significant doubts regarding the vaccine's efficacy. This further highlighted the multilayered nature of vaccine decision-making, which is influenced by regulatory pressure (travel requirements across borders), the mix or circulating correct and wrong information, and transnational consultations and other factors shaping the decision-making toward vaccination among diverse migrant groups. A previous study on infodemics has indicated that information overload from various channels associated with COVID-19 vaccines has negatively impacted the willingness to vaccinate (34), in line with our findings.

The link between COVID-19 infodemic and related stress was studied by Nguyen et al., and they found that the type and reliability of information significantly impacted people's emotional wellbeing (35). Further, health literacy encompasses individuals' abilities to manage emotions and cope with health-related stressors effectively. Our study participants also described this emotional distress, especially with regards to transnational sources of information such as social media platforms including Facebook and WhatsApp. Moreover, this phenomenon could be explained by “pandemic transnationalism” where migrants being exposed to transnational social network and difference in handling the pandemic between country of origin and host country, experienced emotional distress (15). In addition to the emotional due to information overload, the wellbeing of their families back home was also a constant source of emotional distress, exacerbated by the challenging situation in India during the pandemic and their lack of possibility to travel. The social connections and families in migrants' home countries became increasingly crucial amidst the COVID-19 pandemic. With travel restrictions making return visits nearly impossible worldwide, migrants relied heavily on internet, phone calls, and social media to maintain significant aspects of their social lives (14). Understanding the transnational dynamics and cultural contexts of these connections becomes vital for devising effective interventions to communicate health risks during crises.

It is worth noting that all the participants were educated with at least a bachelor's degree. Our participants demonstrated the ability to access, comprehend and apply health information, highlighting a clear link between higher education levels and higher health literacy. However, their transnational ties introduced a layer of complexity to their information landscape, as they navigated health guidelines and updates from both Norwegian and Indian sources, which were sometimes diverge. Thus, we found that health literacy and transnationalism are deeply intertwined, even in the context of infodemics. Health literacy enhances migrants' ability to critically evaluate information from both Norwegian and Indian sources, to take informed health decisions, while transnational influences further shape how migrants' access and interpret health information, underscoring the need for culturally sensitive interventions that strengthen health literacy and empower informed decision-making across diverse informational contexts.

## 5 Strengths and limitations

This study provides the insights into the experiences of participants on how transnational network influence information- and health-seeking behavior during a pandemic, adding depth to the understanding of migrant experiences in Norway, which to our knowledge has not been previously explored. We have worked under the assumption that participants' construct of their experiences are trustworthy, since the objectivity is not independent of a person's consciousness and interpretation of his experiences. Therefore, our interpretation of the data should not be seen as an established truth, but verisimilitude (36) highlighting experience barriers. Further, sample barriers apply, as our sample characteristics may have impacted transferability, that is Indian background, which may not hold true for other migrant groups. However, the detailed descriptions of methodology may provide the ground for transferability. Another potential limitation is the educational barriers that may have influenced our findings, as most participants hold at least a bachelor's degree. This could be considered a weakness, as it might not have adequately captured the perspectives of individuals with basic or no formal education, which could impact digital health literacy and how participants sought health information. Lastly, language barriers were present, as interviews are conducted in English, a non-native language for some participants, causing a language bias, potentially limiting the depth of their responses. Despite these limitations we argue our findings offer important insights that are highly relevant for future research on migrant health information-seeking behavior during pandemics.

Further, credibility in our study was attained by peer debriefing to ensure multiple perspectives on data interpretation, and by demonstrating transparency in reporting the methodology. Reflexivity was maintained through reflective team discussions, allowing researchers to critically examine their own biases and assumptions throughout the research process. Furthermore, our study's relevance could be understood as addressing a timely and important topic, particularly in understanding how migrants navigate health information and practices transnationally during crises, which can inform policies and interventions aimed at improving health communication strategies and future preparedness among migrants.

## 6 Conclusion

Our findings demonstrated that Indian migrants in Norway were particularly affected by the infodemic that accompanied the COVID-19 pandemic, which was characterized by an overwhelming abundance of false or conflicting information. This situation was especially distressing for migrants, who were vulnerable to the sheer volume of misinformation circulating on social media. The study highlighted those migrants relied on trusted sources of information, particularly official government websites in both Norway and India. Additionally, personal networks played a significant role in shaping their health-related decision-making and emotional well-being. Personal experiences shared by family, friends, and neighbors, especially through transnational ties, were considered reliable sources of information, further underscoring the importance of social relationships in health decision-making. Local connections established in Norway, including GPs in both Norway and India, were also perceived as trustworthy sources of guidance amidst conflicting information. The complex interaction between cultural background, transnational ties, and the perceived credibility of these various information sources significantly influenced the decision-making processes and emotional well-being of Indian migrants. Collaboration with migrants to create trust in information and addressing the influence of transnational ties during an infodemic is crucial for developing effective culturally sensitive health communication strategies that support the well-being of migrant populations in global crises. Public health responses should guide migrants to trustworthy information sources, such as community leaders and official health channels in their native languages, to ensure they receive accurate health information. Supporting emotional well-being through culturally appropriate mental health services and peer support groups is essential. Additionally, interventions should consider factors like length of stay, age, and gender, using strategies such as social media outreach for younger migrants and traditional methods for older generations, while also addressing the unique health needs of both men and women in migrant communities. The implications of these findings are crucial for enhancing the effectiveness of public health interventions in multicultural societies, ensuring that migrant populations receive accurate, relevant, and supportive health information during global health crises.

## Data Availability

The datasets discussed in this article are not publicly accessible due to the nature of the research, as participants did not consent to public sharing of their data. However, raw data can be made available upon reasonable request to researchers affiliated with accredited institutions, with necessary redactions to protect confidentiality. Access will be granted to investigators who submit a methodologically sound proposal. To comply with the General Data Protection Regulation (GDPR), data processing must be covered by the European Commission's standard contractual clauses for personal data transfer, which the data requesters must sign. Proposals and data access requests should be directed to the corresponding author. Requests to access the datasets should be directed to Prabhjot Kour, prabhjot.kour@uib.no.
