# Supplementary material for: Living infodemics across borders: experiences during the COVID-19 pandemic among migrants from India living in Norway
Source: Front Public Health. 2025 Feb 28;13:1488080. doi: 10.3389/fpubh.2025.1488080 (PMC11906422; doi:10.3389/fpubh.2025.1488080)
Supplement: Supplementary file 1 [file Data_Sheet_1.docx]

**Appendices**

**Appendix 1**: Table 1

**Table 1: Demographics of participants**

| **Participant ID** | **Age range (in years)** | **Gender** | **Type of education** | **Length of residence in Norway**  **(in years)** |
| --- | --- | --- | --- | --- |
| **P1** | 30-39 | Female | Doctorate | 5 |
| **P2**  **(Dyadic interview with couple)** | 40-49 | Male | Bachelor’s degree | 13 |
|  | 30-39 | Female | Bachelor’s degree | 9 |
| **P3** | 30-39 | Male | Master’s degree | 6 |
| **P4** | 30-39 | Male | Bachelor’s degree | 4 |
| **P5** | 30-39 | Female | Doctorate | 3 |
| **P6**  **(Dyadic interview with couple)** | 20-29 | Female | Bachelor’s degree | 3 |
|  | 30-39 | Male | Master’s degree | 7 |

| **P7** | 40-49 | Male | Bachelor’s degree | 10 |
| --- | --- | --- | --- | --- |
| **P8** | 50-59 | Male | Doctorate | 15 |
| **P9** | 50-59 | Male | High school | 40 |
| **P10** | 70-79 | Male | Bachelor’s degree | 46 |

| **Appendix 2: Interview guide** |
| --- |
| 1. How would you describe the amount and type of information you got on how to handle the |
| pandemics in Norway and in your home country? |
| 2. What were your experiences with COVID-19 information sources during COVID-19 pandemic? |
| - What sources of information did you trust? |
| - How did you share disease information with your colleagues/relatives using social media? |
| - Following COVID-19 information from home country |
| - Health information in Norway |
| - Social media (WhatsApp, Facebook) |
| - Information from friends and relative |
| 3. If there were any, what were the situations that made you confused/panic in this pandemic? |
| - Contradicting information about measures to prevent COVID-19 |
| - Information around COVID-19 vaccine |
| - Medication use |
| - COVID-19 risk perception to oneself and family |
| 4. If you had any such problem, tell me what bothered you most in this pandemic in terms of disease |
| knowledge? |
| - COVID-19 cases in home country |
| - Life after covid |
| - Anxiety because of lack of guidance or information |
| - Trust issues |
| 5. How did you handle any uncertainty regarding information during the pandemic? |
| - Use of GP in Norway |
| - Contact doctor in home country by telephone |
| - Use of pharmacy in Norway |
| - Other |
| 6. What are your thoughts around Norwegian Health authority COVID-19 pandemic updates? |
| - Disease awareness programs |
| - Vaccination follow-up |
| - Regular alerts |
| - Any other |
| 7. Did you have contact with your GP regarding anything related to COVID-19 and if so, can you |
| describe your experience? |
| - Was it an Indian GP? |
| - Was it used for information, treatment, other (like sick leave) |
| - Challenges? |
